# ‘It's like the bad guy in a movie who just doesn't die’: a qualitative exploration of young people's adaptation to eczema and implications for self‐care†

**DOI:** 10.1111/bjd.18046

**Published:** 2019-07-28

**Authors:** D. Ghio, I. Muller, K. Greenwell, A. Roberts, A. McNiven, S.M. Langan, M. Santer

**Affiliations:** ^1^ Primary Care and Population Science, Faculty of Medicine University of Southampton Southampton U.K; ^2^ Centre for Clinical and Community Applications of Health Psychology Faculty of Social and Human Sciences University of Southampton Southampton U.K; ^3^ Centre of Evidence Based Dermatology School of Medicine University of Nottingham Nottingham U.K; ^4^ Nuffield Department of Primary Care Health Sciences Radcliffe Observatory Quarter Woodstock Road Oxford OX2 6GG U.K; ^5^ Faculty of Epidemiology & Population Health London School of Hygiene and Tropical Medicine London U.K; ^6^ Health Data Research U.K. London U.K

## Abstract

**Background:**

Eczema is a common childhood inflammatory skin condition, affecting more than one in five children. A popular perception is that children ‘outgrow eczema’, although epidemiological studies have shown that, for many, eczema follows a lifelong episodic course.

**Objectives:**

To explore the perceptions of young people about the nature of their eczema and how these perceptions relate to their self‐care and adapting to living with eczema.

**Methods:**

This is a secondary inductive thematic analysis of interviews conducted for Healthtalk.org. In total 23 interviews with young people with eczema were included. Of the 23 participants, 17 were female and six male, ranging from 17 to 25 years old.

**Results:**

Participants generally experienced eczema as an episodic long‐term condition and reported a mismatch between information received about eczema and their experiences. The experience of eczema as long term and episodic had implications for self‐care, challenging the process of identifying triggers of eczema flare‐ups and evaluating the success of treatment regimens. Participants’ experiences of eczema over time also had implications for adaptation and finding a balance between accepting eczema as long term and hoping it would go away. This linked to a gradual shift in treatment expectations from ‘cure’ to ‘control’ of eczema.

**Conclusions:**

For young people who continue to experience eczema beyond childhood, a greater focus on self‐care for a long‐term condition may be helpful. Greater awareness of the impact of early messages around ‘growing out of’ eczema and provision of high‐quality information may help patients to manage expectations and support adaptation to treatment regimens.

**What's already known about this topic?**

- There is a common perception that people ‘grow out of’ eczema, but for many people eczema follows a lifelong episodic course.
- Qualitative work has shown that parents can find that being told their child will grow out of eczema is dismissive, and that they have difficulty with messages about ‘control not cure’ of eczema.
- It is unclear how young people perceive their eczema and the implications of this perception for their adaptation and self‐care.

**What does this study add?**

- The message that many people ‘grow out of’ eczema has a potentially detrimental effect for young people where the condition persists.
- This has implications for young people's perceptions of their eczema, their learning to self‐care and how they adapt to living with eczema and eczema treatments.

**What are the clinical implications of this work?**

- Clinicians need to promote awareness among young people that eczema is a long‐term episodic condition in order to engage them with effective self‐care.
- Young people transitioning to self‐care need evidence‐based information that is specific and relatable to them.

## Abstract 2

https://doi.org/10.1111/bjd.18649 available online

https://www.bjdonline.com/article/

Eczema is a common condition that typically starts within the first 2 years of life, recurring episodically throughout childhood. It is often expected to improve by adolescence.^1^ However, epidemiological evidence shows that eczema frequently persists into adolescence^2^ or adulthood, and is better viewed as a lifelong episodic condition.^3^, ^4^ In this study we use the term ‘eczema’ to refer to ‘atopic eczema’ (synonymous with ‘atopic dermatitis’).^5^

The view of eczema as a condition that is likely to resolve by adolescence may have implications for how people with eczema and their families perceive the condition. Qualitative research has shown that parents of children with eczema can find assurances that their child will ‘grow out of it’ as a ‘fobbing off’ or dismissal of the seriousness of the condition.^6^, ^7^ Furthermore, viewing eczema as a childhood condition rather than a longer‐term condition may contribute to families’ discomfort with messages about treatments aiming to ‘control’ not ‘cure’ the condition,^6^, ^8^ with implications for their adherence to long‐term time‐consuming treatments such as applying emollients.

Existing research therefore suggests the message that ‘eczema will resolve’ may influence how people perceive advice and approaches to self‐care of the condition. It is also possible that such messages influence how people with eczema and their families ‘adapt’ to the condition. In the unified theory, Moss‐Morris^9^ postulates that successful adaptation to a long‐term condition requires adjustment of both cognitive (e.g. beliefs and attitudes) and behavioural (e.g. adherence to regimens) factors. According to the unified theory, adaptation subsequently leads to positive outcomes in distress, good illness management and less interference in life.

For adolescents and emerging adults, adaptation would occur during a time where the management of a condition becomes a shared task, transferring primary responsibility from the family and parents to the young person.^10^ Adolescence and emerging adulthood is a time for developing individual social identity and autonomy from parents.^11^ Learning to take responsibility or share responsibility for the long‐term condition therefore comes at a time where they are also seeking autonomy, which may have an impact on adjustment and self‐care.

Little qualitative research has been carried out among children with eczema. Wishes for a ‘miracle cure’ may be linked to the view of eczema as a condition that is likely to resolve.^12^ We are not aware of similar work carried out among teenagers or young adults, although one qualitative study found that adolescents’ attitudes can range from indifferent to more negative dependent on the severity of their eczema.^13^ However, it is unclear how this perception links to how they adjust to having eczema. The aim of this study was to explore the perceptions of young people about the nature of their eczema and how these perceptions relate to self‐care of the condition and living with the condition.

## Patients and methods

### Design and data collection

The study was a secondary analysis of qualitative interview data.^14^ The qualitative interviews were originally carried out for the Healthtalk SKINS project, which aimed to explore the information and support needs of young people with four common skin conditions: acne, eczema, psoriasis and alopecia. Interview extracts were published on the multimedia website, Healthtalk.org, as either written, video or audio clips. This paper is based on a reanalysis of full transcripts to explore experiences of young people around self‐care of eczema.

In total 97 semistructured interviews were carried out with people aged 13–25 years with skin conditions in England. The term ‘young people’ is used to describe this sample age group. Participants were recruited from dermatology departments, general practice clinics, social media, dermatology charities’ mailing lists, universities and schools. Of these interviews, 24 were with young people with eczema. A.M. carried out the interviews between October 2014 and December 2015. All but one interviewee consented to a secondary analysis. Interviews were transcribed verbatim and checked by participants for accuracy.

More details regarding the recruitment and data collection process can be found in McNiven.^15^ The Healthtalk.org project was approved by Berkshire NRES Committee South Central, and the secondary analysis was approved by Wales REC 7 Ethics Committee (REC 17/WA/0329). To recruit a diverse sample across patients with all skin conditions A.M. created a sampling matrix,^16^ which took into consideration aspects such as age, sex, ethnicity, geographical location and study or employment status.^15^ The interview schedule included topics of diagnosis experience, early knowledge and information, treatment and management, everyday life with a skin condition, sharing information with other young people with eczema, changes over time and anticipated future. The interviews took place in different settings preferred by the participants such as their homes or local community centres and lasted up to 2 h.

### Analysis

The data were coded using inductive methodology of latent thematic analysis^17^ in NVivo software (version 11; QSR International, Doncaster, Australia). Participants were identified using pseudonyms.

All codes were derived from the data, and no preidentified theoretical frameworks or codebooks were used. Author D.G. completed immersion in the data by reading the full transcripts and then coded nine transcripts. A draft coding manual was developed and then discussed with the analysis team (I.M., M.S., K.G.). Two transcripts were also coded by the team to test the draft coding manual. Following discussions, changes were made to the coding manual and a further three transcripts were coded with the coding manual to ensure that codes were identified and defined as clearly as possible. Triangulation within the team provided different perspectives and interpretations to expand understanding of the data. To maximize credibility, the team checked all coded data against the definitions in the coding manual to ensure the data matched the agreed definition and then to identify representative quotes.^17^

Once all of the transcripts were coded, the analysis team met to discuss the data as we sought to summarize themes from related codes. Data analysis identified four core themes. These were (i) barriers to and facilitators of treatment, (ii) transition to self‐management, (iii) symptoms related to eczema and (iv) the episodic nature of eczema. There was a prominent recurring core theme about the episodic nature of eczema that ran throughout the young people's accounts. We recognized that most of the young people had their first diagnosis in early childhood and that their current understanding of eczema was in the context of the duration of their condition. This led to the development of an analysis plan to explore how experiences of eczema throughout participants’ lifespans influenced their perceptions of the condition. This paper focused on this core theme regarding the episodic nature of eczema. Theory relevant to adapting to long‐term conditions was then explored to develop a diagram of theoretical framework to understand how the codes relate to each other.

## Results

There were 23 text transcripts of interviews with young people with eczema who had given permission for secondary analyses. Of the 23 participants, 17 were female and 15 had had eczema for their whole life. The mean age was 21 years (range 17–25).

**Figure 1:**
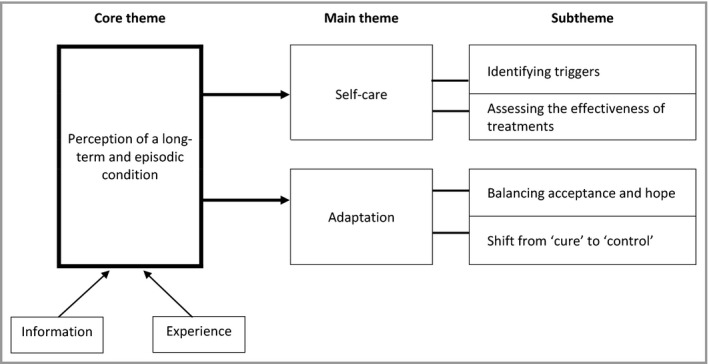
The developed thematic framework where young people perceived or experienced eczema as a long‐term episodic condition. This had implications for two main themes: self‐care and adaptation to eczema, which included identifying triggers and effective treatment, and acceptance and shifting treatment expectations.

We identified a core theme running throughout the interviews: that all participants perceived eczema as a long‐term and episodic condition. This perception was usually informed by many years’ experience of eczema, and generally did not match the message they had often been given, usually by health professionals, that their eczema would resolve or improve. From this overarching core theme, we developed two main themes: (i) self‐care of a long‐term and episodic condition and (ii) adaptation to a long‐term and episodic condition. Each of these main themes has two subthemes (Fig. 1).

### Perceptions of eczema as a long‐term and episodic condition

Young people said that, through their experience, they had come to perceive eczema as a long‐term and episodic condition. They described their eczema ‘going away’; sometimes this meant that their symptoms improved, sometimes this referred to the eczema having resolved. They also spoke about how their eczema varied over their lifespan. There were periods of their lives where eczema was ‘always coming back’ or flaring up, which led participants to conclude that their eczema would never fully go away.

‘It feels like it will never go away even though I've had like, I think I've had about a couple of years free from it since I was a child [laughs], it's always coming back and it sort of comes back with a vengeance and it's just like, ah it's like the bad guy in a movie who just doesn't die.’ Dua, age 21 years (female)

This perception of the long‐term nature of eczema was generally at odds with information they had received, as many of the interviewees spoke of being told in childhood that they would ‘grow out of’ eczema. Some spoke about this as a general belief about eczema and some said this information came directly from their health professionals. This information contradicted their experiences because all the participants continued to get eczema flare‐ups into adolescence. Some had spoken about how they had been asymptomatic for a time and then felt disappointment when eczema recurred.

‘Everyone just thinks you grow out of it, which I thought I had. […] That's sort of quite difficult especially when you had that period without it and then suddenly it sort of comes back, […] everyone's sort of perception is that everyone, you just grow out of it, and a lot of people do but at the same time it can just sort of come back.’ Lanie, age 24 years (female)

When participants talked about their memories of having eczema as a child, it was evident that they tried to make sense of the information they had been given about eczema and how it related to their experiences. Some young people felt that eczema was often presented as a childhood condition, therefore implying that it would end after childhood. Other young people used concrete examples as evidence of how ‘growing out of’ eczema was not reflective of their experiences. One person explained how their father still had eczema and this led her to question whether she would grow out of eczema. Some participants were told by different sources (general practitioner and internet) about a 7‐year cycle where the skin changes every 7 years, but this also did not match with their experiences.

‘It's all kind of very random with me. I don't know if the 7‐year cycle theory has any sort of backing […] obviously the 7 years that I was sort of under 7 weren't too bad, and then when I was 7–14 it did get a little bit worse, and now kind of 14–21 and coming to the end of like a calm period. I don't know, it would make sense but I think again you're just thinking about it and you can just apply it to yourself, it's like clutching at straws really.’ Vikki, age 20 years (female)

### Implications for self‐care of the episodic nature of eczema

We found that the episodic nature of eczema had implications for two different aspects of self‐care: identifying what triggers a flare‐up and assessing the effectiveness of different treatment approaches. Most participants were still seeking the causes of their flare‐ups and trying to make sense of their experiences and treatments, despite having had eczema since childhood.

#### Identifying triggers

Causes of eczema were perceived as multidimensional, potentially changing and evolving over time. During eczema flare‐ups, participants tried to identify what might have triggered this. Suspected triggers included increased stress, hormonal changes and/or puberty, alcohol, weather and moving to different cities, suggesting a link between life events or other environmental factors and their eczema. However, the episodic nature of the condition contributed to uncertainty around causation of a flare‐up.

‘It was really bad when I was a child and then it kind of died down for a bit and then I suppose when I got to being a teenager and did kind of use different creams and stuff on my skin and like cosmetics and whatever and shaving my legs then it did flare up again I suppose. But I wasn't sure, it's difficult to say what that's related to, it could be like hormones or and then it's flared up again more recently in the last few years as well so, I don't know, I think my skin just goes through cycles.’ Bridget, age 22 years (female)

#### Assessing the effectiveness of treatments

The episodic nature of eczema also meant that it was difficult for young people to assess whether an improvement or deterioration in their eczema was due to changes in their treatment plan or due to the episodic nature of the condition.

‘Mum would sort of try out different foods, different creams, different treatments, and it's a very much like a cycle sort of skin condition […] as much as you try different things, you know you never know whether it's, which factor has improved it or made it worse, so it's a bit of a trial and error.’ Charlotte, age 22 years (female)

Furthermore, when young people were not experiencing any eczema symptoms, they described that there was no motivation or need to continue engaging in any help or information‐seeking behaviours.

‘I'd be interested in trialling any, any new kind of creams or steroids or, or treatments that would be out there. [erm] I haven't heard of any. But that's not to say I haven't, well I haven't looked at all. [erm] I've just been happy because over the past couple of months to a year that my skin's generally been quite good. And so you, you automatically forget to think about these kind of things. And you only think about it when you have a flare‐up.’ Mali, age 23 years (male)

### Implications for adaptation to the long‐term and episodic nature of eczema

Learning to live with the fluctuating and long‐term nature of eczema was related to a young person's adaptation to the condition. Overall, participants spoke about changes over time to how they adjusted to eczema, learnt how to live with it, and learnt to apply what they knew about controlling their eczema.

‘Well since I was born with it I've learnt to accept…like I've learnt to live with it so, I couldn't really give you any experiences, it's something just…I honestly don't know […] cos it's always been part of me. I've never experienced having no eczema, so for me this is normal you know. And then you get people going, “Oh what's it like to have eczema then?” I'm like, “Well it's a bit…well, you know it's a pain but you know you just learn to live with it.” And I've always, always struggled with it, so but as I've got older I've learnt to control it more.’ Gabi, age 17 years (female)

This adjustment was seen in two subthemes: how they struggled between accepting eczema as a long‐term condition while hoping it ‘goes away’, and the need to shift from expecting treatment will cure eczema to expecting the treatment will control eczema.

#### Striking a balance between acceptance and hope

Many participants described uncertainty about whether or not their eczema would have an impact on them in the long term. Some young people dealt with this uncertainty through accepting that their eczema was likely to be long term, while others continued to hope that it would go away. Both of these coping approaches had implications for self‐care. Successful adaptation required both hope and acceptance. Hoping that eczema would eventually improve motivated young people to seek and continue to seek effective self‐care strategies to find resolution for their eczema.

‘Get it over with, it's not unmanageable. And I always get to like 90% healed and then 10% is left and I'm like, “OK, it's fine now.” And then that 10% always strikes back so I need to squash out the 10% as well.’ Zaahira, age 21 years (female)

On the other extreme, some participants described accepting a level of disruption with little hope that their eczema would resolve. This type of acceptance was also described alongside a disengagement in help‐seeking behaviours.

‘I talk to my parents about it but recently I've just kind of got used to it. Which is kind of bad but I have. [um] And it's kind of dealing with it. So, trying to deal with it mentally but I'm getting round it […] I didn't really tell my teachers. I just got used to the pain so I just kept, I had to keep going with writing or whatever I was doing.’ Tom, age 17 years (male)

Another example of acceptance of eczema as a long‐term condition was present in those interviewees who spoke about the impact of eczema on their confidence and how this changed over time. Many participants described adapting to the challenges of having a visible skin condition and learning to be more confident and accept it as part of who they were. These young people overcame their self‐consciousness by acceptance of eczema as part of their everyday life and identity. Some of the participants were resistant to the notion that eczema was a defining identifier, showing there is a balance between accepting a condition as being part of their identity and it not being the complete identity.

‘I don't really know, I just kind of accepted it as part of who I was and what I had to do, and cos it…at times it like affected you daily; it was just part of what you did. There wasn't really a point when I was like, “Oh this is what I have to live with,” like it wasn't like a dawning realization; I think I just sort of got on with it but, that is largely part of who I am [laughs] so, it was never really like a, “Oh no” moment.’ Fearne, age 21 years (female)

#### Shift from seeking ‘cure’ to ‘control’ of eczema

As part of adapting and accepting eczema treatment as part of their daily lives, there was a shift from young people perceiving treatments as a means of curing eczema to a means of keeping eczema under control. People spoke about learning how to manage eczema, including applying their treatment, as having become part of their routine or habits.

‘I'm hopeful that will like clear up my eye eczema. […] But then I think, I guess like the biggest thing I could hope for is that like, I know it's not gonna get cured, it's not something that's gonna go away probably for, it might like be able to manage it.’ Willow, age 23 years (female)

## Discussion

Young people experienced and perceived eczema as a long‐term and episodic condition. This perception and experience influenced their self‐care of eczema because its episodic course made it harder to identify triggers of flare‐ups or effective treatments. Furthermore, this perception also influenced the young people's beliefs and attitudes about treatment and acceptance of the condition. The young people's experiences were challenged by the information they had been given about eczema being a ‘childhood condition’, which contradicted their personal experiences of eczema, providing an added barrier to self‐care and adaptation to living with the condition. This highlighted the importance of information provided during childhood.

Usually, at the stage prior to self‐care, it is often the parents who would receive the information and take responsibility for managing the condition. As the young person gets older they often do not receive information again. Therefore, there is a need to provide specific information about the whole condition (e.g. cause, duration) directed at the young person and not their parents or carers regarding eczema prognosis and treatment, especially as they transition to taking responsibility for their self‐care.

Research with adults with hand eczema and with psoriasis has identified unmet needs in terms of information and support in self‐management of episodic skin conditions.^18^, ^19^ This gap in support may partly explain why adolescents may create their own treatment routines, which differ from guidelines provided by healthcare providers.^13^ There is an unmet need for more information and support for self‐care in eczema, as evident in work with carers,^6^, ^20^, ^21^ and our findings suggest that young people transitioning to self‐care may need even greater support.

This lack of accurate information reflecting their experiences means that people with long‐term conditions may not feel supported in addressing the disruption such a condition creates.^22^, ^23^ Attempts to try to gain control by making sense of the condition, including identification of patterns in cause and effect in order to predict and manage the symptoms, may reflect coping mechanisms.^24^ However, adults with hand eczema described frustration with the unpredictability of fluctuations and difficulty identifying triggers.^18^

Young people with eczema experienced similar challenges and stressors to young people with other long‐term conditions, especially when the condition posed a threat to their social identity.^25^, ^26^ Over time, for some young people this led to benefit finding, a key behavioural response where the individual tries to identify a positive perspective in a situation.^9^ Many young people shift from perceiving this struggle of being different to strengthening their self‐confidence.

These findings concur with research regarding adaptation to long‐term conditions. The unified theory postulates that acceptance is an important factor to successful adjustment, and that coping through wishful thinking leads to adjustment difficulties.^9^ Our findings suggest that young people's acceptance of eczema, and their hope that it will go away, are in conflict with each other. They tend to know there is no cure for eczema but continued to hope it would go away. In a review of qualitative work with children and young people with long‐term conditions, there were similar themes of striving for acceptance and adjusting with everyday treatment regimens as part of their everyday lives.^26^ For example, children with asthma eventually accept suboptimal control through striving to feel ‘normal’.^27^ When young people refer to having a condition as their norm, it is because they are shifting from comparing themselves with their peers, to comparing with their own experiences of the condition. They compare the now self with their past self with the same symptoms.^28^

Redefining normalcy can be a way to deal with the concept of biographical disruption, which for young people is a disruption of the process in creating an identity.^28^ However, an added challenge is highlighted in the findings of the current study, where young people with episodic conditions experience time with and without the symptoms of the condition. The changing and evolving nature of eczema make redefining normalcy even more difficult. This all occurs in the context of transitioning into adulthood, where young people may experience developmental challenges including those related to long‐term conditions.^29^

A limitation of the current study is that the sample included young people who mostly had eczema since childhood, and the sample included only six male participants. The groups were too small to be able to explore in detail how the duration and severity of eczema or familial prior knowledge or experience of eczema contributed to changes in beliefs and behavioural responses. The relationships between perceptions of eczema and self‐care are theorized in this paper; further work will be required to understand implications and to determine how perceptions of eczema impact on adaption and self‐care. As this was a secondary data analysis there was no scope for carrying out additional interviews to explore these themes further. This may have given a more in‐depth understanding of the impact of the episodic nature of eczema. However, this is one of the first qualitative analyses to explore how young people make sense of having eczema and gives us insight into the experiences of young people with persistent forms of childhood conditions.

In conclusion, our findings show that key messages communicated in childhood have long‐term implications for how young people respond to living with eczema and long‐term use of eczema treatments. Support for self‐care needs to shift from treating eczema as a childhood condition to considering eczema as a long‐term episodic condition for those who continue to experience eczema. This means there is a need to provide knowledge that is relevant for young people with eczema and their families to develop realistic expectations to support them in adapting and adhering to treatment routines.

## Supporting information


**Powerpoint S1** Journal Club Slide Set.

## References

[1] Williams HC . Clinical practice. Atopic dermatitis. N Engl J Med 2005; 352:2314–24. 15930422 10.1056/NEJMcp042803

[2] Margolis JS , Abuabara K , Bilker W , et al. Persistence of mild to moderate atopic dermatitis. JAMA Dermatol 2014; 150:593–600. 24696036 10.1001/jamadermatol.2013.10271 PMC4352328

[3] Abuabara K , Yu AM , Okhovat JP *et al* The prevalence of atopic dermatitis beyond childhood: a systematic review and meta‐analysis of longitudinal studies. Allergy 2018; 73:696–704. 28960336 10.1111/all.13320 PMC5830308

[4] Weidinger S , Novak N . Atopic dermatitis. Lancet 2016; 387:1109–22. 26377142 10.1016/S0140-6736(15)00149-X

[5] Johansson SGO , Bieber T , Dahl R *et al* Revised nomenclature for allergy for global use: report of the Nomenclature Review Committee of the World Allergy Organization, October 2003. J Allergy Clin Immunol 2004; 113:832–6. 15131563 10.1016/j.jaci.2003.12.591

[6] Santer M , Burgess H , Yardley L *et al* Experiences of carers managing childhood eczema and their views on its treatment: a qualitative study. Br J Gen Pract 2012; 62:261–7. 10.3399/bjgp12X636083 PMC3310032 22520913

[7] Gore C , Johnson RJ , Caress AL *et al* The information needs and preferred roles in treatment decision‐making of parents caring for infants with atopic dermatitis: a qualitative study. Allergy 2005; 60:938–43. 15932385 10.1111/j.1398-9995.2005.00776.x

[8] Smith SD , Hong E , Fearns S *et al* Corticosteroid phobia and other confounders in the treatment of childhood atopic dermatitis explored using parent focus groups. Australas J Dermatol 2010; 51:168–74. 20695854 10.1111/j.1440-0960.2010.00636.x

[9] Moss‐Morris R . Adjusting to chronic illness: time for a unified theory. Br J Health Psychol 2013; 18:681–6. 24118260 10.1111/bjhp.12072

[10] Newbould J , Smith F , Francis S‐A . ‘I'm fine doing it on my own’: partnerships between young people and their parents in the management of medication for asthma and diabetes. J Child Health Care 2008; 12:116–28. 18469296 10.1177/1367493508088549

[11] Sroufe LA , Cooper RG , Marshall ME . Child Development – Its Nature and Course. New York: McGraw‐Hill, 1988.

[12] Wake EV , Batchelor J , Lawton S *et al* The views of children and young people on the use of silk garments for the treatment of eczema: a nested qualitative study within the CLOTHing for the relief of Eczema Symptoms (CLOTHES) randomized controlled trial. Br J Dermatol 2018; 178:183–90. 28856661 10.1111/bjd.15909 PMC6487959

[13] Kosse RC , Bouvy ML , Daanen M *et al* Adolescents’ perspectives on atopic dermatitis treatment: experiences, preferences, and beliefs. JAMA Dermatol 2018; 154:824–7. 29847623 10.1001/jamadermatol.2018.1096 PMC6128492

[14] Ziebland S , Hunt K . Using secondary analysis of qualitative data of patient experiences of health care to inform health services research and policy. J Health Serv Res Policy 2014; 19:177–82. 24573821 10.1177/1355819614524187

[15] McNiven A . ‘Disease, illness, affliction? Don't know’: ambivalence and ambiguity in the narratives of young people about having acne. Health (London) 2019; 23:273–88. 29552892 10.1177/1363459318762035

[16] Coyne IT . Sampling in qualitative research. Purposeful and theoretical sampling; merging or clear boundaries? J Adv Nurs 1997; 26:623–30. 9378886 10.1046/j.1365-2648.1997.t01-25-00999.x

[17] Braun V , Clarke V . Using thematic analysis in psychology. Qual Res Psychol 2006; 3:77–101.

[18] Mollerup A , Johansen JD , Thing LF . Knowledge, attitudes and behaviour in everyday life with chronic hand eczema: a qualitative study. Br J Dermatol 2013; 169:1056–65. 23889257 10.1111/bjd.12524

[19] Khoury LR , Skov L , Møller T . Facing the dilemma of patient‐centred psoriasis care: a qualitative study identifying patient needs in dermatological outpatient clinics. Br J Dermatol 2017; 177:436–44. 28032892 10.1111/bjd.15292

[20] Teasdale EJ , Muller I , Santer M . Carers’ views of topical corticosteroid use in childhood eczema: a qualitative study of online discussion forums. Br J Dermatol 2017; 176:1500–7. 27753076 10.1111/bjd.15130

[21] Allen C , Vassilev I , Kennedy A *et al* Long‐term condition self‐management support in online communities: a meta‐synthesis of qualitative papers. J Med Internet Res 2016; 18:e61. 26965990 10.2196/jmir.5260 PMC4807245

[22] Bury M . Chronic illness as biographical disruption. Sociol Health Illness 1982; 4:167–82. 10.1111/1467-9566.ep11339939 10260456

[23] Bury M . The sociology of chronic illness: a review of research and prospects. Sociol Health Illness 1991; 13:451–68.

[24] Burger JM , Arkin RM . Prediction, control, and learned helplessness. J Personality Soc Psychol 1980; 38:482–91.

[25] Davidson M , Penney ED , Muller B *et al* Stressors and self‐care challenges faced by adolescents living with type 1 diabetes. Appl Nurs Res 2004; 17:72–80. 15154119 10.1016/j.apnr.2004.02.006

[26] Lambert V , Keogh D . Striving to live a normal life: a review of children and young people's experience of feeling different when living with a long term condition. J Pediatr Nurs 2015; 30:63–77. 25450440 10.1016/j.pedn.2014.09.016

[27] Ireland LM . Children's perceptions of asthma: establishing normality. Br J Nurs 1997; 6:1059–64. 9370569 10.12968/bjon.1997.6.18.1059

[28] Williams B , Corlett J , Dowell JS *et al* ‘I've never not had it so I don't really know what it's like not to’: nondifference and biographical disruption among children and young people with cystic fibrosis. Qual Health Res 2009; 19:1443–55. 19805806 10.1177/1049732309348363

[29] Sawyer SM , Drew S , Yeo MS *et al* Adolescents with a chronic condition: challenges living, challenges treating. Lancet 2007; 369:1481–9. 17467519 10.1016/S0140-6736(07)60370-5

