# Supplementary material for: ‘It's like the bad guy in a movie who just doesn't die’: a qualitative exploration of young people's adaptation to eczema and implications for self‐care
Source: Br J Dermatol. 2019 Jul 28;182(1):112–8. doi: 10.1111/bjd.18046 (PMC6972719; doi:10.1111/bjd.18046)
Supplement: Supplementary file 1 — Powerpoint S1 Journal Club Slide Set. [file BJD-182-112-s001.pptx]

## Slide 1
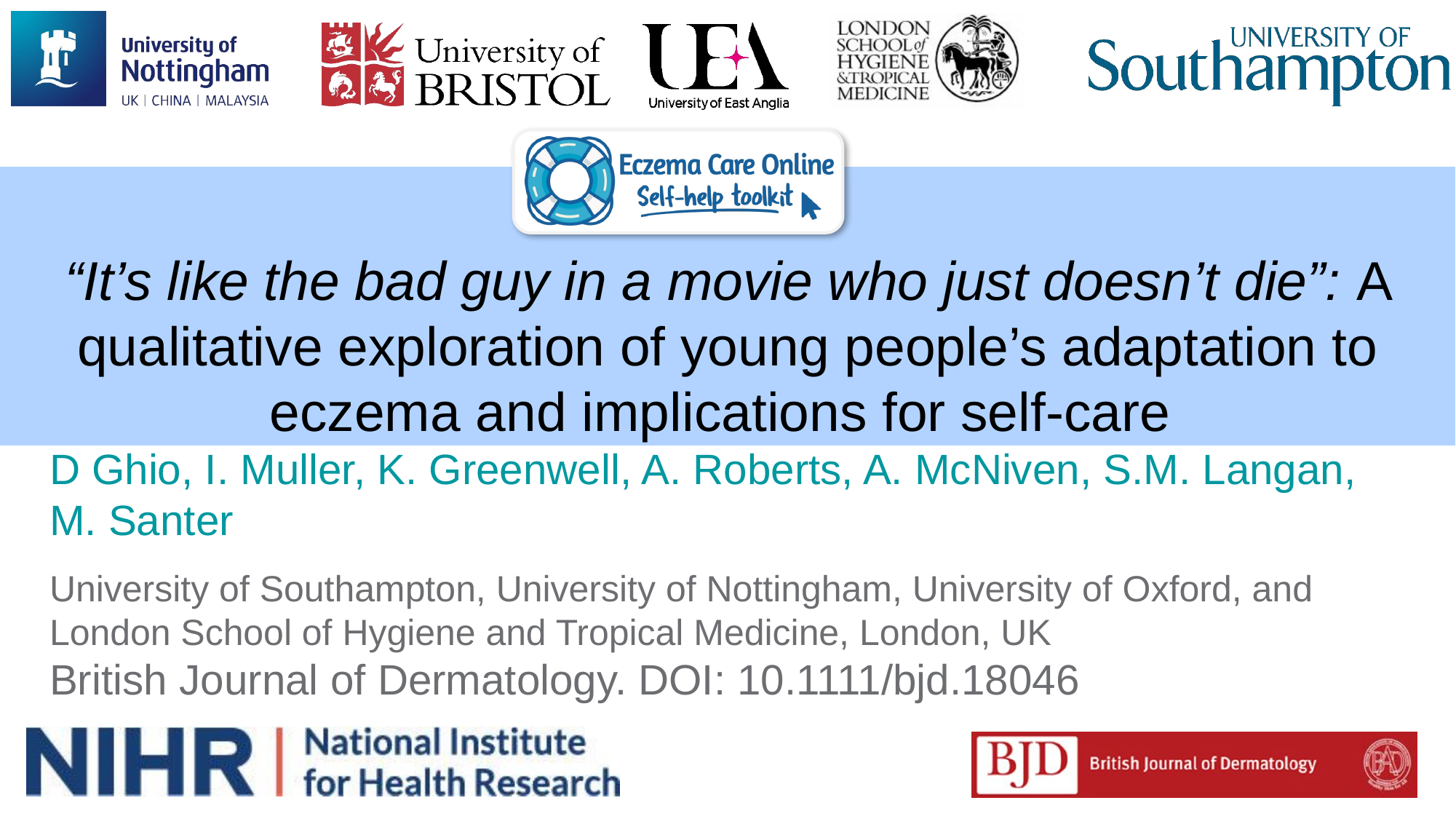

# “It’s like the bad guy in a movie who just doesn’t die”: A qualitative exploration of young people’s adaptation to eczema and implications for self-care
D Ghio, I. Muller, K. Greenwell, A. Roberts, A. McNiven, S.M. Langan, M. Santer
University of Southampton, University of Nottingham, University of Oxford, and London School of Hygiene and Tropical Medicine, London, UK
British Journal of Dermatology. DOI: 10.1111/bjd.18046

## Slide 2
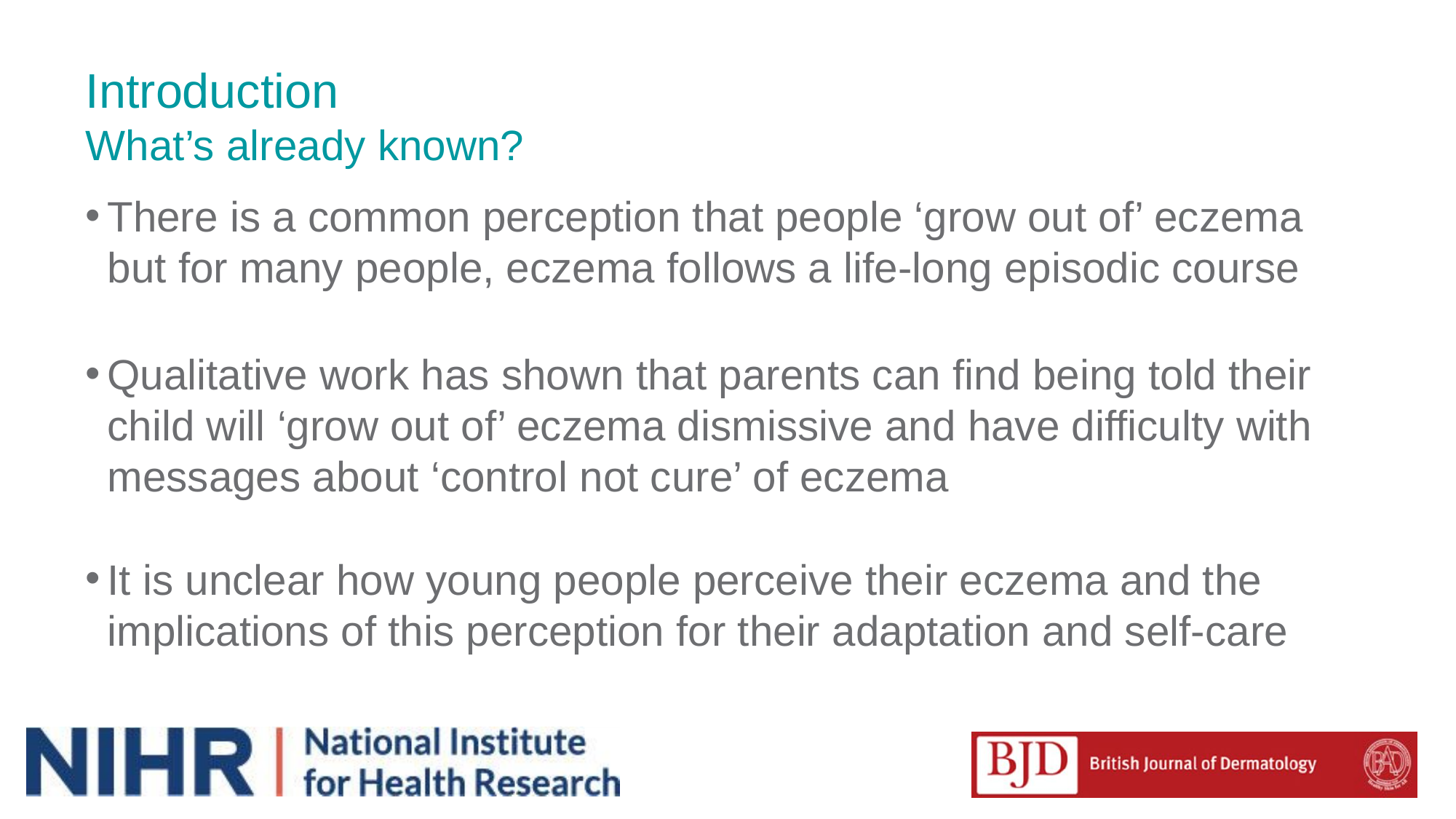

# Introduction What’s already known?
There is a common perception that people ‘grow out of’ eczema but for many people, eczema follows a life-long episodic course
Qualitative work has shown that parents can find being told their child will ‘grow out of’ eczema dismissive and have difficulty with messages about ‘control not cure’ of eczema
It is unclear how young people perceive their eczema and the implications of this perception for their adaptation and self-care

## Slide 3
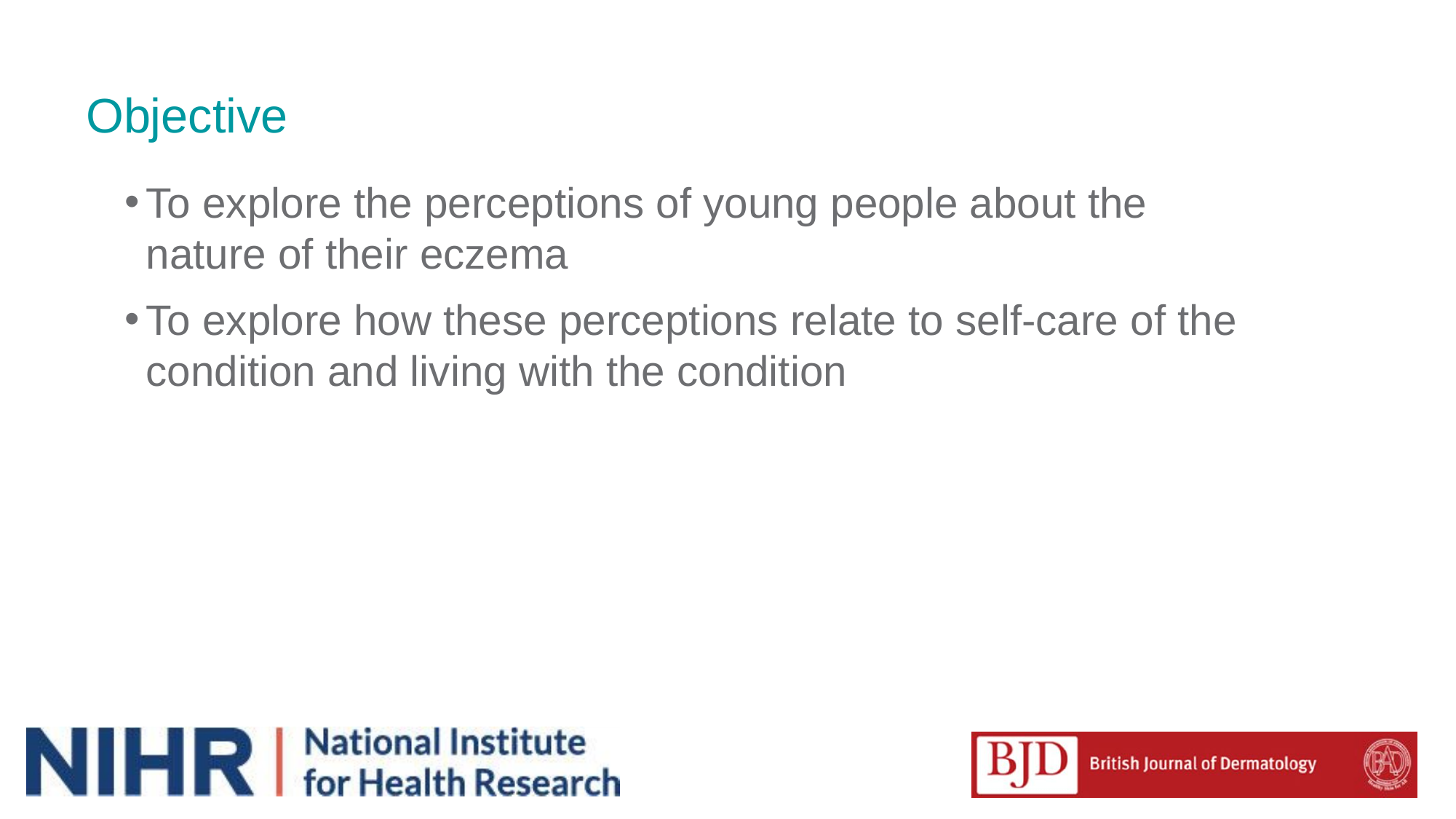

# Objective
To explore the perceptions of young people about the nature of their eczema
To explore how these perceptions relate to self-care of the condition and living with the condition

## Slide 4
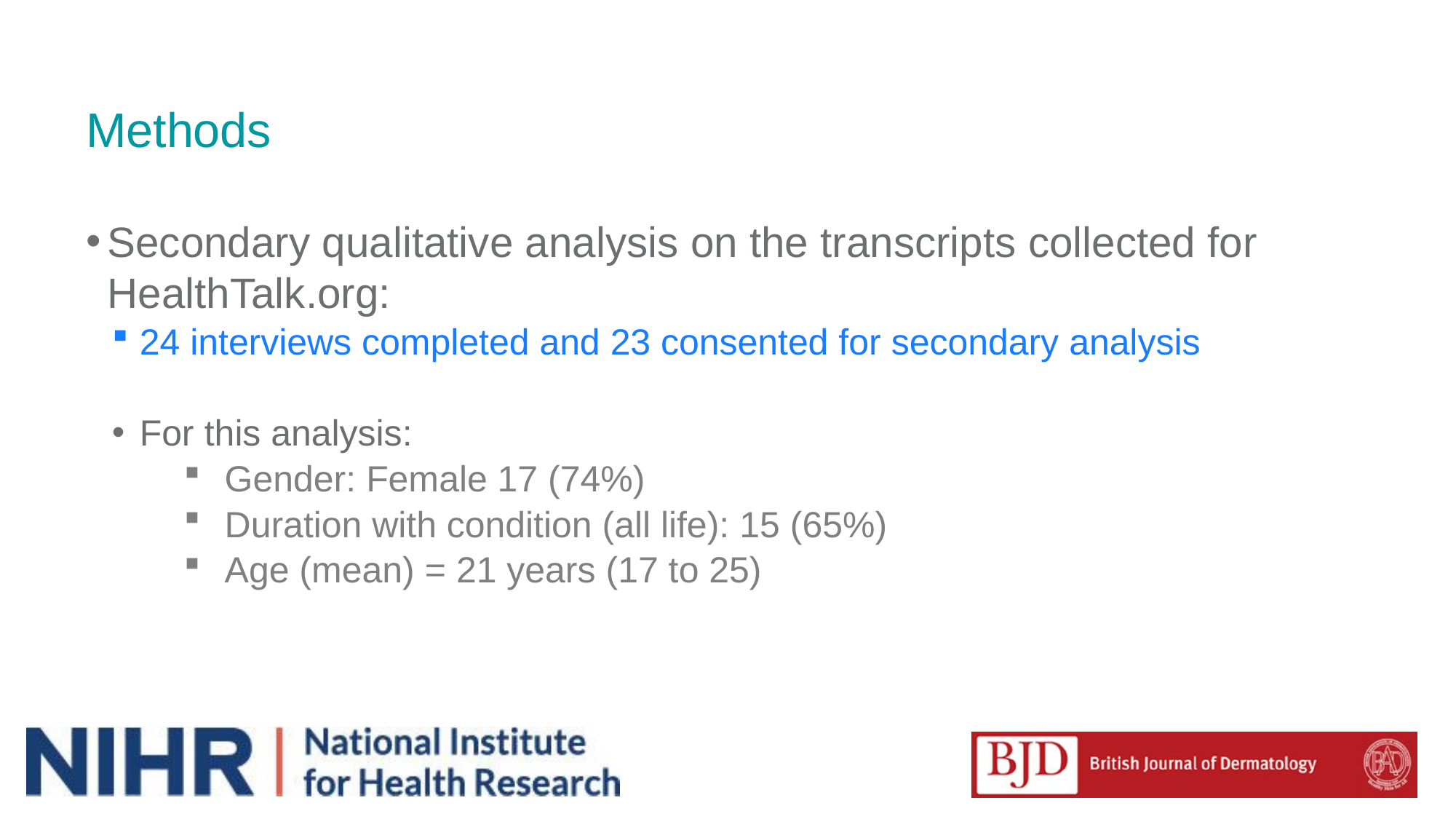

# Methods
Secondary qualitative analysis on the transcripts collected for HealthTalk.org:
24 interviews completed and 23 consented for secondary analysis
For this analysis:
Gender: Female 17 (74%)
Duration with condition (all life): 15 (65%)
Age (mean) = 21 years (17 to 25)

## Slide 5
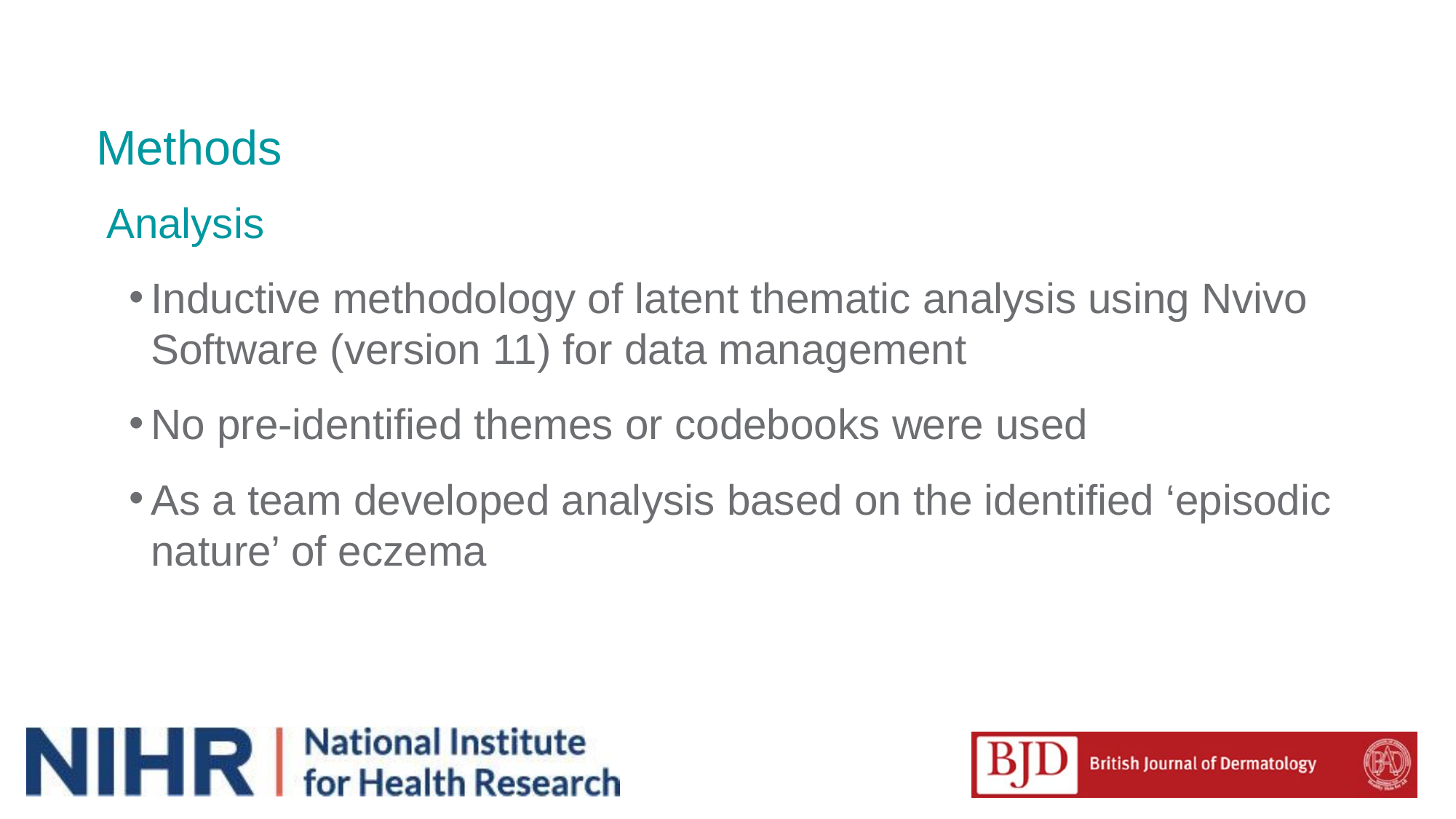

# Methods Analysis
Inductive methodology of latent thematic analysis using Nvivo Software (version 11) for data management
No pre-identified themes or codebooks were used
As a team developed analysis based on the identified ‘episodic nature’ of eczema

## Slide 6
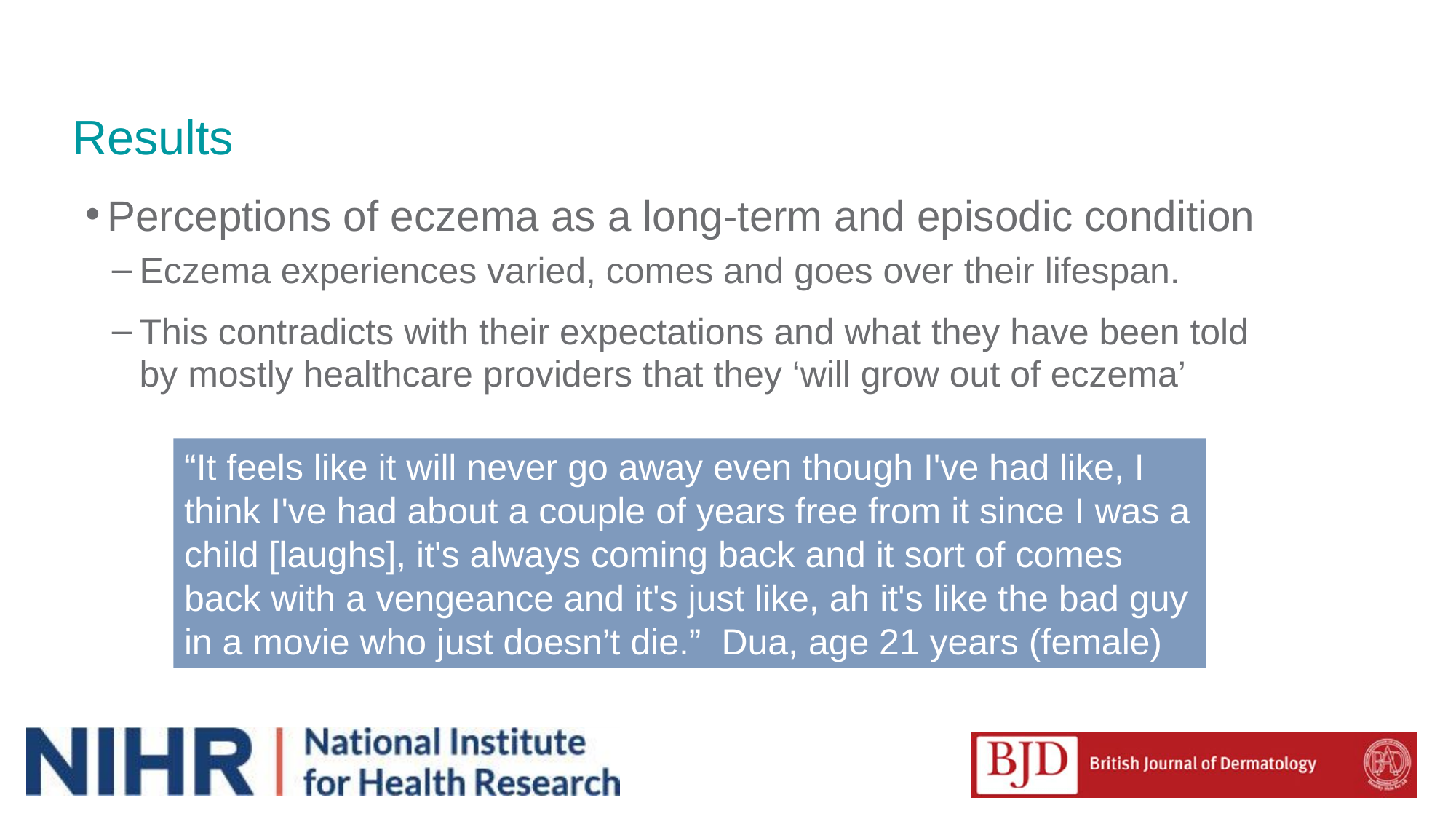

# Results
Perceptions of eczema as a long-term and episodic condition
Eczema experiences varied, comes and goes over their lifespan.
This contradicts with their expectations and what they have been told by mostly healthcare providers that they ‘will grow out of eczema’
“It feels like it will never go away even though I've had like, I think I've had about a couple of years free from it since I was a child [laughs], it's always coming back and it sort of comes back with a vengeance and it's just like, ah it's like the bad guy in a movie who just doesn’t die.” Dua, age 21 years (female)

## Slide 7
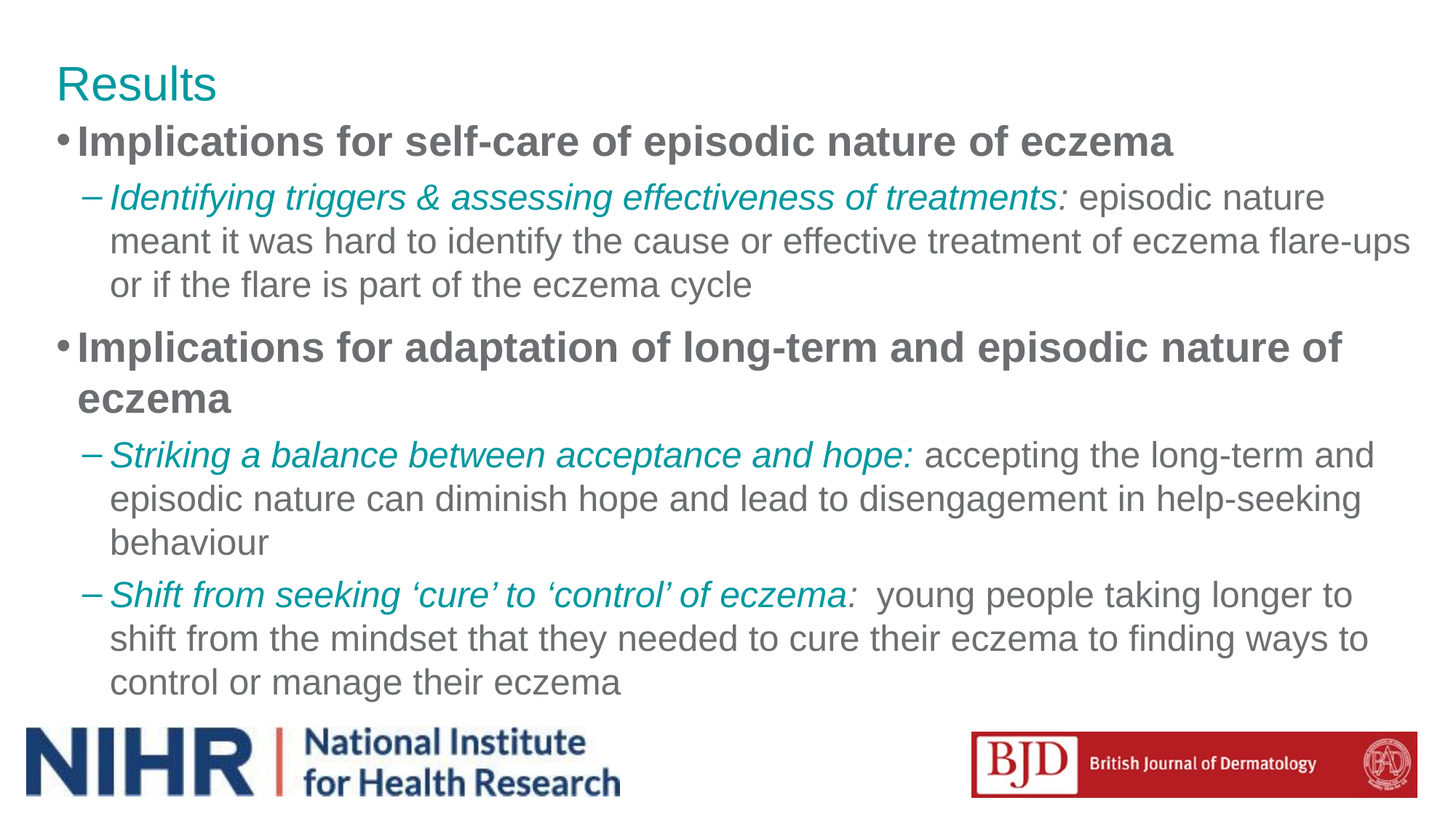

# Results
Implications for self-care of episodic nature of eczema
Identifying triggers & assessing effectiveness of treatments: episodic nature meant it was hard to identify the cause or effective treatment of eczema flare-ups or if the flare is part of the eczema cycle
Implications for adaptation of long-term and episodic nature of eczema
Striking a balance between acceptance and hope: accepting the long-term and episodic nature can diminish hope and lead to disengagement in help-seeking behaviour
Shift from seeking ‘cure’ to ‘control’ of eczema: young people taking longer to shift from the mindset that they needed to cure their eczema to finding ways to control or manage their eczema

## Slide 8
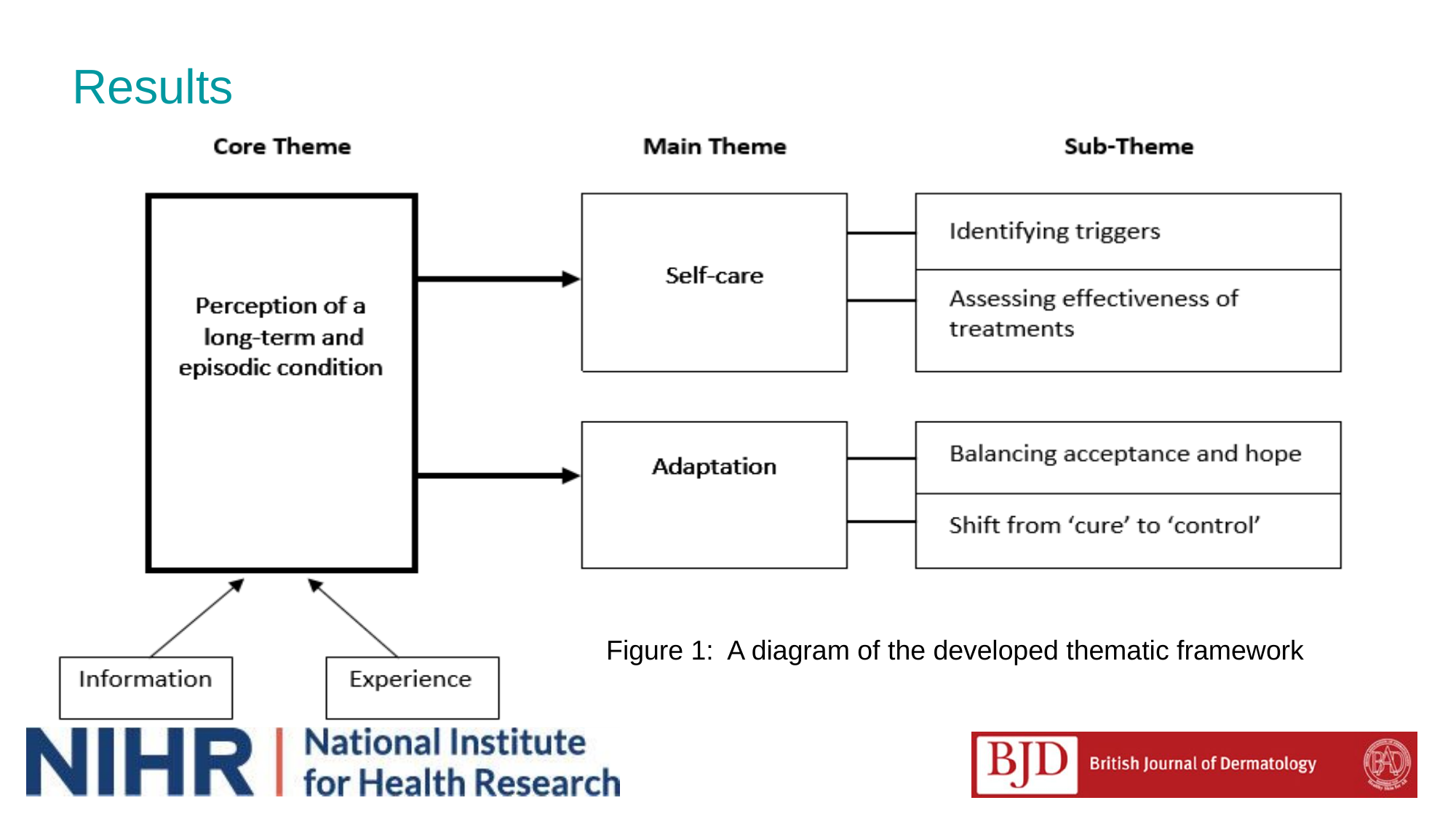

# Results
Figure 1: A diagram of the developed thematic framework

## Slide 9
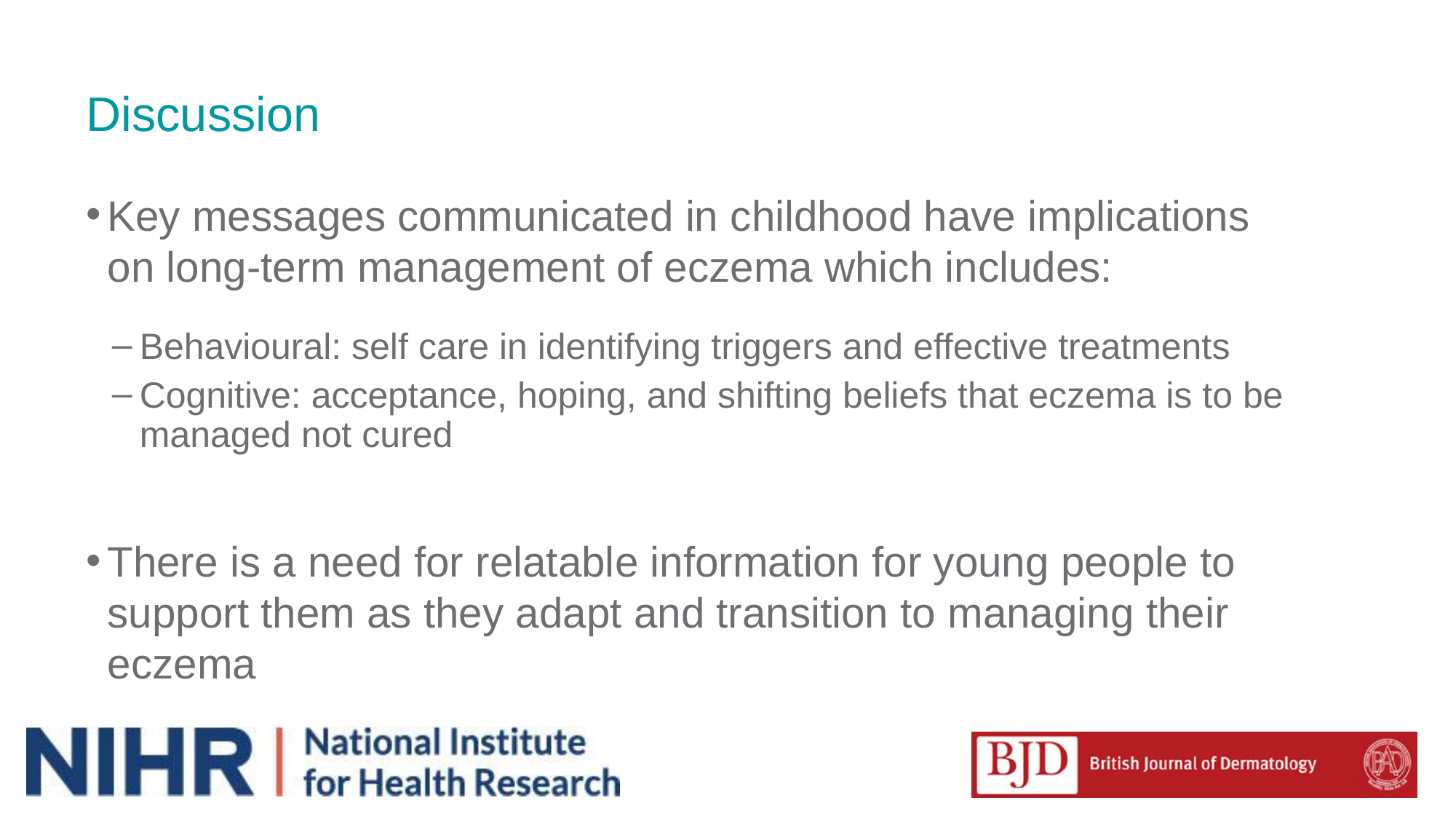

# Discussion
Key messages communicated in childhood have implications on long-term management of eczema which includes:
Behavioural: self care in identifying triggers and effective treatments
Cognitive: acceptance, hoping, and shifting beliefs that eczema is to be managed not cured
There is a need for relatable information for young people to support them as they adapt and transition to managing their eczema

## Slide 10
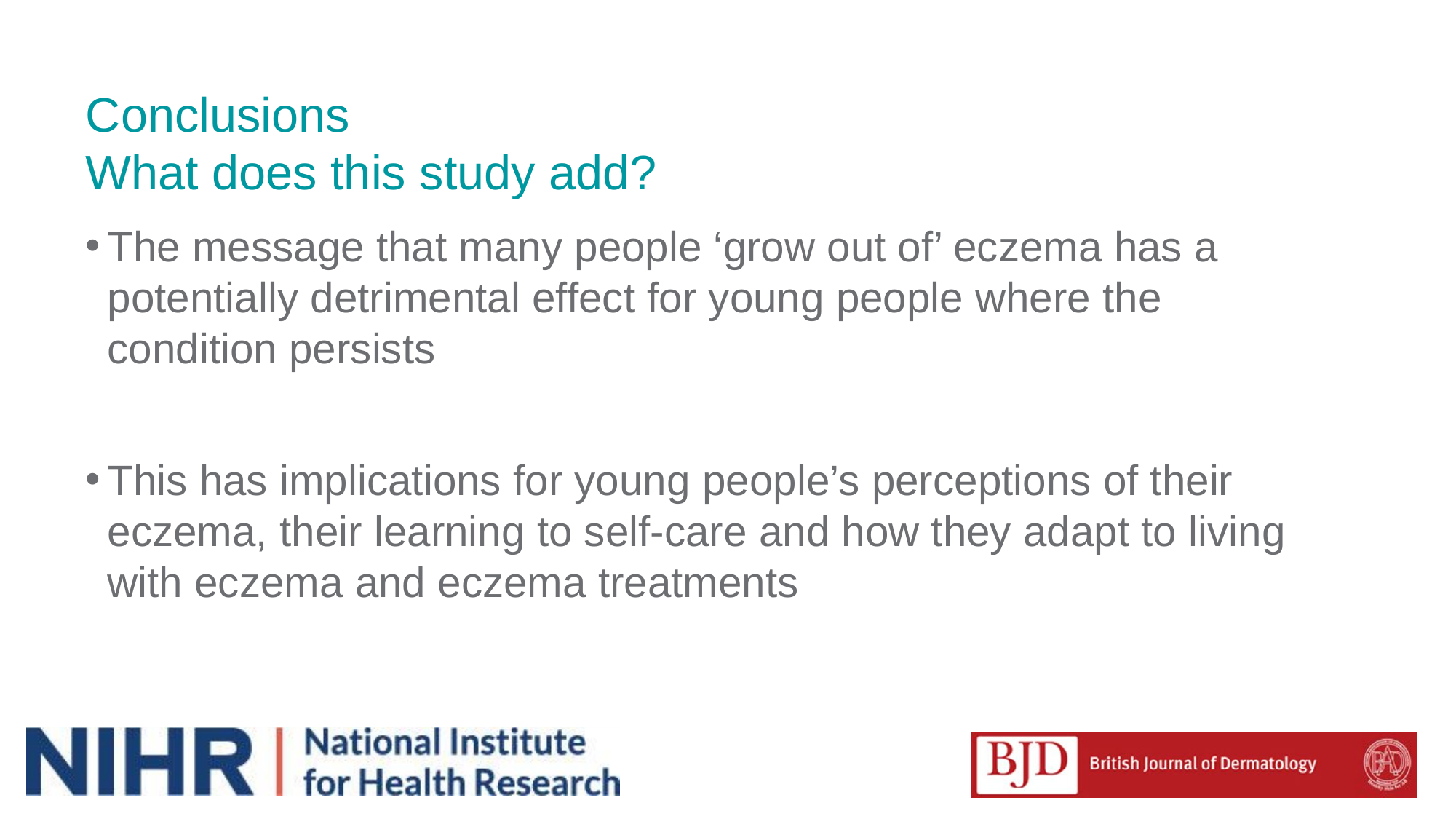

# Conclusions What does this study add?
The message that many people ‘grow out of’ eczema has a potentially detrimental effect for young people where the condition persists
This has implications for young people’s perceptions of their eczema, their learning to self-care and how they adapt to living with eczema and eczema treatments

## Slide 11
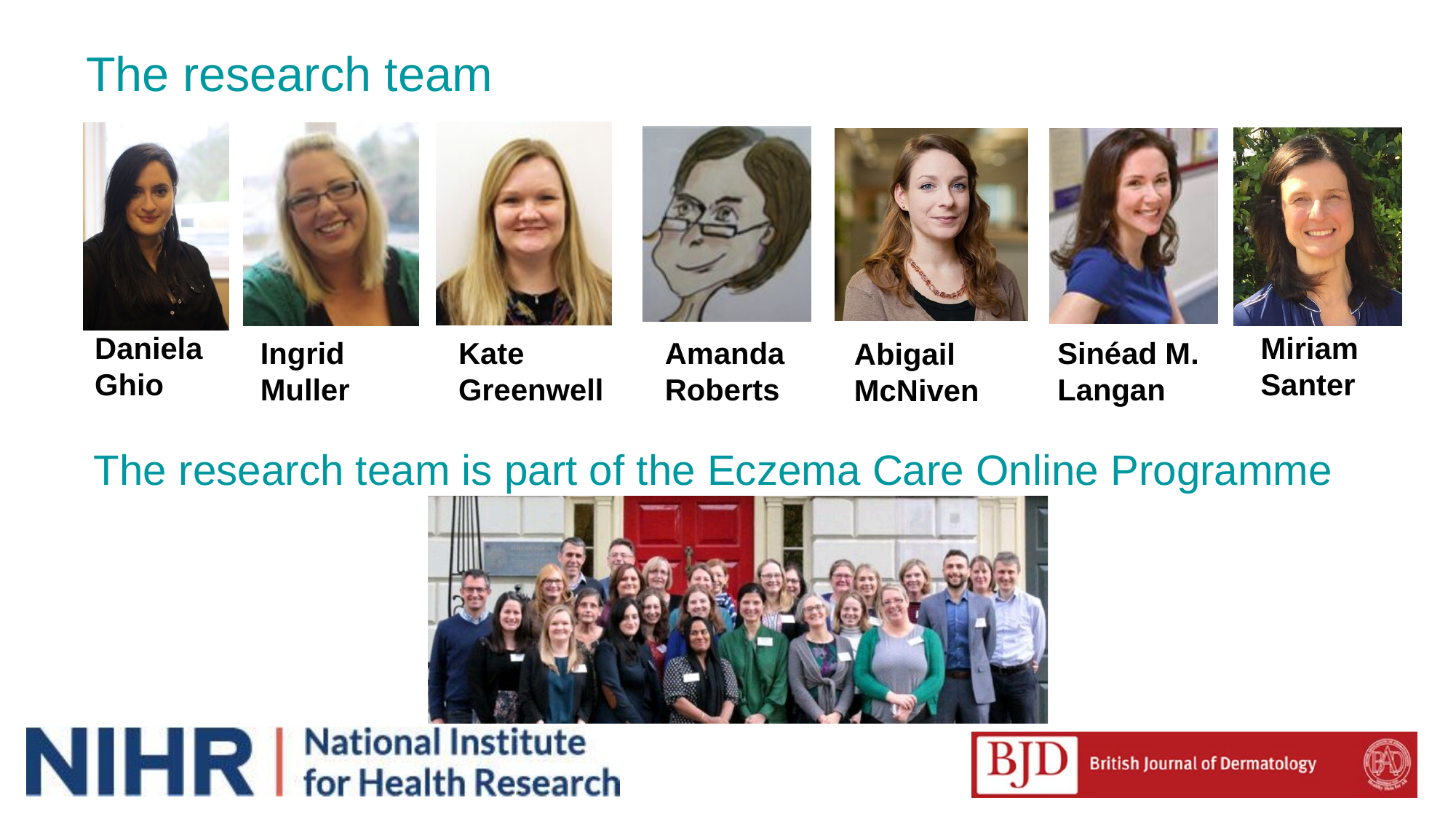

# The research team
Daniela Ghio
Miriam Santer
Ingrid Muller
Kate Greenwell
Amanda Roberts
Sinéad M. Langan
Abigail McNiven
The research team is part of the Eczema Care Online Programme

## Slide 12
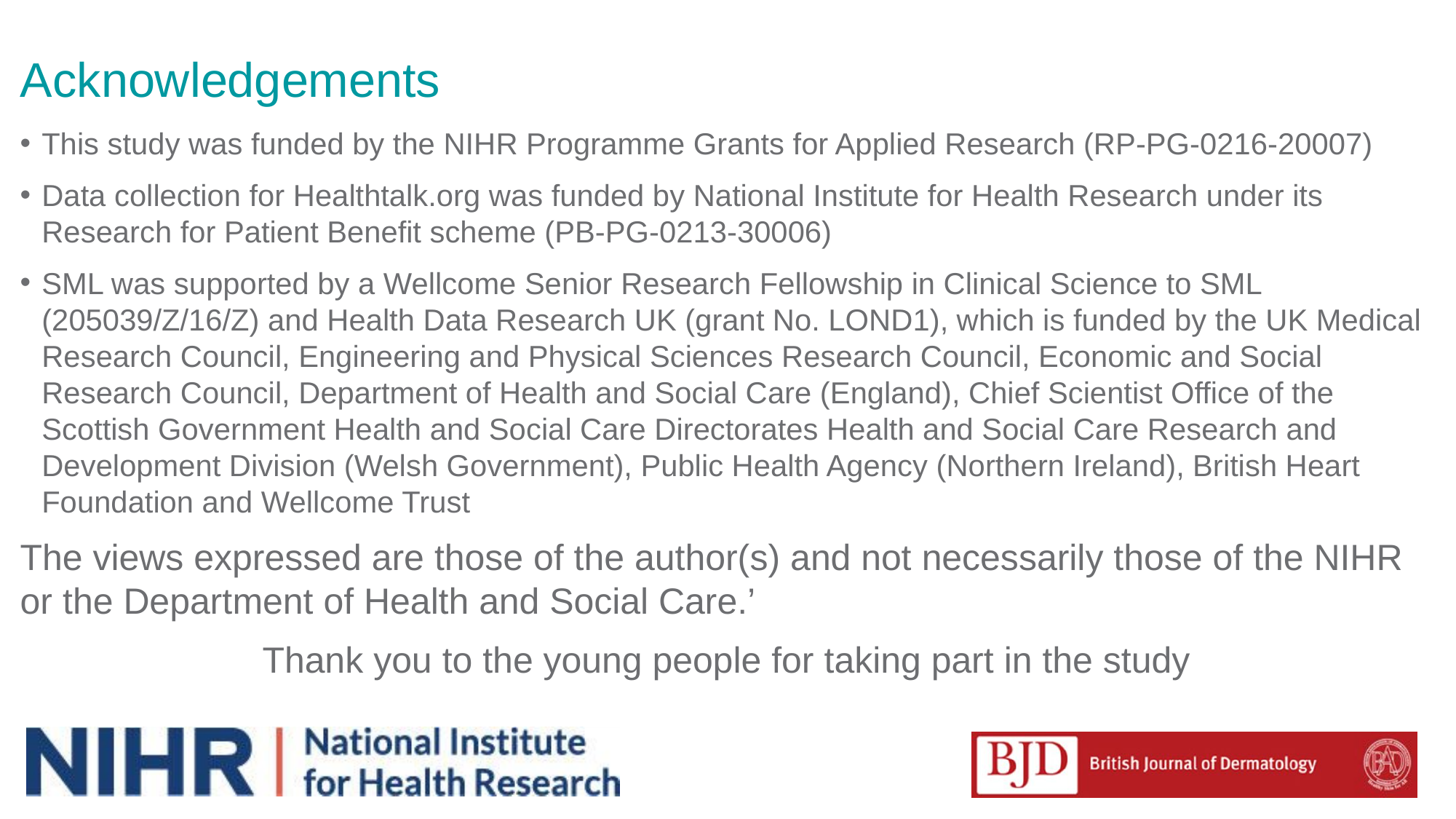

# Acknowledgements
This study was funded by the NIHR Programme Grants for Applied Research (RP-PG-0216-20007)
Data collection for Healthtalk.org was funded by National Institute for Health Research under its Research for Patient Benefit scheme (PB-PG-0213-30006)
SML was supported by a Wellcome Senior Research Fellowship in Clinical Science to SML (205039/Z/16/Z) and Health Data Research UK (grant No. LOND1), which is funded by the UK Medical Research Council, Engineering and Physical Sciences Research Council, Economic and Social Research Council, Department of Health and Social Care (England), Chief Scientist Office of the Scottish Government Health and Social Care Directorates Health and Social Care Research and Development Division (Welsh Government), Public Health Agency (Northern Ireland), British Heart Foundation and Wellcome Trust
The views expressed are those of the author(s) and not necessarily those of the NIHR or the Department of Health and Social Care.’
Thank you to the young people for taking part in the study

## Slide 13
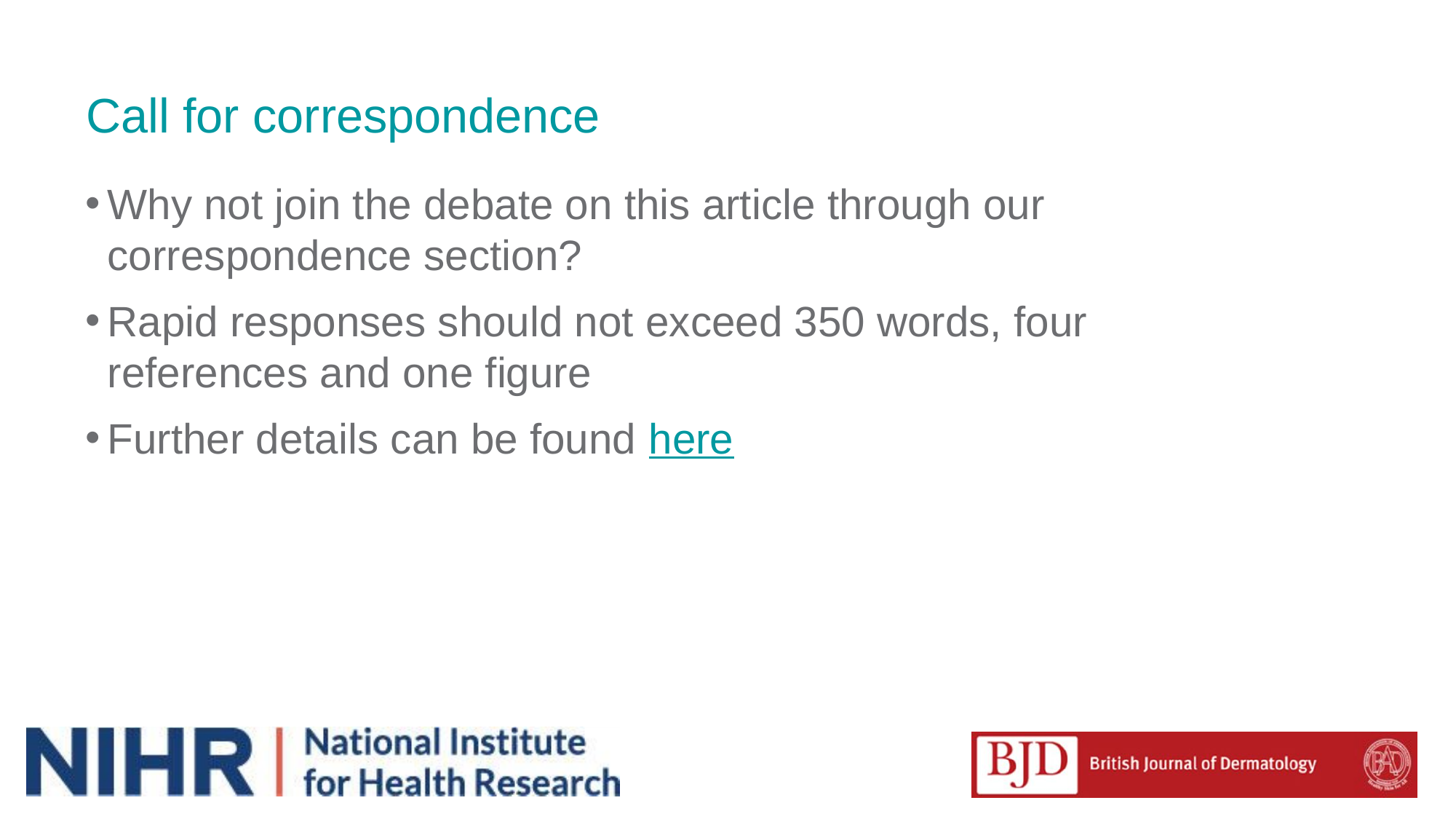

# Call for correspondence
Why not join the debate on this article through our correspondence section?
Rapid responses should not exceed 350 words, four references and one figure
Further details can be found here
